# Pediatric Acute-Onset Neuropsychiatric Syndrome (PANS) and Pediatric Autoimmune Neuropsychiatric Disorders Associated with Streptococcal Infections (PANDAS): Immunological Features Underpinning Controversial Entities

**DOI:** 10.3390/children11091043

**Published:** 2024-08-27

**Authors:** Lucia Leonardi, Camilla Perna, Irene Bernabei, Marco Fiore, Meiqian Ma, Jennifer Frankovich, Luigi Tarani, Alberto Spalice

**Affiliations:** 1 Department of Maternal Infantile and Urological Sciences, Sapienza University of Rome, Viale del Policlinico 155, 00161 Rome, Italy; 2 Institute of Biochemistry and Cell Biology, IBBC-CNR, 00185 Rome, Italy; 3 Division of Allergy, Immunology & Rheumatology, Department of Pediatrics, Stanford University School of Medicine, Palo Alto, CA 94305, USA

**Keywords:** PANDAS, PANS, immunological features, IL-17, autoantibodies, autoimmune diseases

## Abstract

Pediatric acute-onset neuropsychiatric syndrome (PANS) and Pediatric Autoimmune Neuropsychiatric Disorders Associated with Streptococcal Infections (PANDAS), represent an overlapping group of disorders which is characterized by acute-onset obsessive compulsive disorders, eating restriction, tics, cognitive and behavioral deterioration which typically follows a relapsing-remitting course but some patients have a primary or secondary persistent progress. This condition is likely caused by heterogeneous inflammatory mechanisms (autoantibodies, complement activation, pro-inflammatory cytokine production) involving the basal ganglia as evidenced by imaging studies (patients vs. controls), sleep studies that found movements and/or atonia during REM sleep, and neurological soft signs that go along with basal ganglia dysfunction. The condition causes significant psychiatric and behavioral symptoms, caregiver burden and sleep abnormalities. Autoantibodies resulting from molecular mimicry of infectious agents (namely group A *Streptococcus*) and neuronal autoantigens that map to the basal ganglia play also a subtle role. This narrative review aims to describe the key immunological features documented thus far and that likely play a role in the pathogenesis and clinical manifestations of this disorder.

## 1. Introduction

Annually, Group A β-hemolytic Streptococcus (GABHS) is responsible for acute pharyngitis in more than 600 million children worldwide [1]. High levels of morbidity in GABHS-related illness can result from direct infection or immune-mediated disease including acute rheumatic fever (ARF), glomerulonephritis and neurological disorders [2,3]. Among the latter, movement disorders (Sydenham’s chorea (SC), tics, hyperactivity, and motor stereotypies) and psychiatric disorders, such as obsessive compulsive disorder (OCD) are described [4]. In 1998 the Pediatric Autoimmune Neuropsychiatric Disorders Associated with Streptococcal Infections (PANDAS) were first described as being characterized by an abrupt onset of OCD and/or tics associated with other neuropsychiatric symptoms related to repeated GABHS infections [5]. PANDAS diagnoses are based on a clinical evaluation and defined by specific criteria (Table 1). 

Similarly to SC, post-infectious immune-mediated pathogenesis has been considered for PANDAS [6]. As in SC, many cases of PANDAS present after the window of opportunity to detect GABHS; thus, operationally, the PANDAS criteria fail to be useful in the clinical setting, although it is a helpful research classification. In SC, the criteria do not require evidence of recent GABHS (neither throat culture nor GABHS antibodies). In 2012, a novel disorder—PANS (Pediatric Acute-onset Neuropsychiatric Syndrome)—was described in order to (1) capture cases where the opportunity to detect GABHS had passed; (2) be agnostic regarding the preceding infection since it is impossible in individual cases to prove causality; (3) open up the possibility that other infections may play a role in triggering the neuropsychiatric deterioration; and (4) add abrupt-onset eating restriction to abrupt-onset OCD as a key symptom [7]. The new PANS criteria left off “tics” as a primary criterion in order to distinguish it from Tourette’s which may also be an immunologically driven condition [7,8], clinical manifestations cannot be better explained by other disorders such as SC, or Tourette syndrome (TS) (all of which tend to have a sub-acute onset) (Table 1), though it is clear that these conditions have significant overlap in symptoms and neurological manifestations thus it is up to the clinician to choose the category which best fits the case. 

The mean age at onset of neuropsychiatric symptoms in PANS patients is 6–9 years [5,7,9,10,11,12]. In addition to GABHS, other microorganisms have been reported at PANS onset (*Mycoplasma pneumoniae*, *Borrelia burgdorferi*, *Staphylococcus aureus*, *Epstein–Barr virus*, *Influenza virus*, *Coxsackie virus* and *Varicella-Zoster virus*); however, the role of GABHS in these cases may have been missed if the patients presented after the window of opportunity to detect GABHS. Environmental factors such as oxidative toxin exposure or psychophysical stress have been proposed as possible triggers of PANS [7,13,14]. PANDAS is not a subset of PANS; rather, it is an overlapping condition since both define an overlapping subset of cases where a key feature is the abrupt onset of OCD.

**Table 1:** PANDAS diagnostic criteria and PANS diagnostic criteria.

1998 PANDAS Criteria [5]	2012 PANS Criteria [7]
(1) Presence of diagnosis of OCD and/or tic disorder	(1) Abrupt, dramatic onset of obsessive compulsive disorder or severely restricted food intake.
(2) Pediatric onset (symptoms first evident between age 3 and the beginning of puberty)	(2) Concurrent presence of additional neuropsychiatric symptoms, with a similarly severe and acute onset, from at least two of the following categories: Anxiety; Emotional lability and/or depression; Irritability, aggression, and/or severely oppositional behaviors; Behavioral (developmental) regression; Deterioration in school performance; Sensory or motor difficulties; Somatic signs or symptoms, including sleep disturbances, enuresis, or urinary frequency.
(3) Episodic course (characterized by abrupt onset of symptoms or dramatic symptom exacerbations)	(3) Symptoms are not better explained by a known neurologic or medical disorder, such as Sydenham chorea, systemic lupus erythematosus, Tourette syndrome, or others.
(4) Association with group A beta-hemolytic *Streptococcus* infection.	
(5) Association with neurologic abnormalities	

PANS/PANDAS are both characterized by abrupt-onset OCD and are followed by a relapsing/remitting course [10,15]. In some cases, the syndrome could cause a gradual deterioration of cognitive function [16]. Four brain imaging studies [17,18,19] point towards the basal ganglia as the most prominently affected part of the brain (which goes along with the symptoms) with symptoms including swelling of the basal ganglia in the acute stage [17]; microglia activation most prominently affects the basal ganglia structures in the caudate and putamen [18], and two studies have indicated microstructural changes in the basal ganglia which may go along with inflammation and/or injury [19,20]. Similar findings are seen in brain MRI studies of SC [21]. Additionally, three studies demonstrated a high prevalence of movements or lack of atonia during REM sleep [22,23,24], which also strongly points towards dysfunction in the basal ganglia as these sleep issues predict Parkinson’s disease (a known basal ganglion disorder) in adults. Additionally, a study found that 22% of patients with PANS have a positive glabellar tap, another finding that predicts Parkinson’s which typically disappears in infancy [25].

A multifactorial etiopathogenesis is assumed for PANS and PANDAS, characterized by an interaction between environmental and genetic factors. A whole-exome sequencing analysis on 386 PANS patients and whole-genome sequencing on 10 severe PANS patients was conducted by Trifletti; in 21 patients candidate mutations (de novo or ultra-rare) were found in 11 genes converging into peripheral regulation of immune responses, microglia, and neuronal synaptic function [26]. 

Immune-mediated pathogenesis is suspected in PANS/PANDAS. It is hypothesized that acute infections or other triggers induce an immune response mediated by interleukin (IL) 17 and other cytokines that disrupt the blood–brain barrier (BBB), as demonstrated in a mouse model [27] and other neuroinflammatory disorders [28]. Cross-reactive autoantibodies which bind to neuronal targets in the basal ganglia are thought to play a role in PANDAS like in ARF/SC [29,30,31,32,33,34,35,36]. The persistence of inflammatory activity is also thought to predispose to a relapsing/remitting disease course [37] as is true with multiple sclerosis.

**Figure 1:**
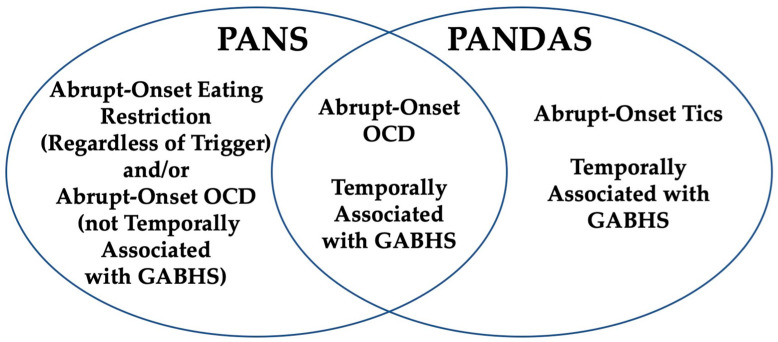
PANS/PANDAS overlapping criteria. GABHS = Group A *Streptococcus* Infection. PANS criteria are agnostic to infection but cases may be temporally associated with GABHS. PANDAS Criteria require that the condition is temporarily associated with GABHS; however, these criteria miss cases since patients often present after the window of opportunity to detect GABHS. These criteria are good for research but not practical for clinical care. Patients with abrupt-onset tics (when associated with GABHS) may satisfy PANDAS criteria but not PANS criteria. Patients with abrupt-onset eating restriction (regardless of the infectious trigger) satisfy PANS criteria but not PANDAS criteria. Both conditions are generally relapsing/remitting. PANS criteria require at least 2 other abrupt-onset neuropsychiatric symptoms in addition to OCD and or eating restriction but PANDAS criteria do not require adjunct symptoms. The PANS and PANDAS criteria strongly overlap since abrupt-onset OCD and preceding GABHS are highly prevalent in cohorts of patients meeting these criteria.

Figure 1 shows the overlapping conditions for the deciphering of PANS/PANDAS. Patients presenting with a sudden onset of tics may fulfill the diagnostic criteria for PANDAS if there is a confirmed signal of a preceding *Streptococcus* infection; however, these individuals would not meet the criteria for PANS due to the precise symptom requirements. On the other hand, individuals who experience an abrupt beginning of eating restrictions associated with *Streptococcus* exposure, but who do not display OCD symptoms, would qualify for a diagnosis of PANS, yet they would not meet the diagnostic criteria for PANDAS.

The similarity between the two syndromes appears evident in individuals who experience an unexpected onset of OCD that is temporally associated with *Streptococcus* exposure; these patients fulfill the diagnostic criteria for both PANS and PANDAS. This peculiarity underscores the nuanced alterations in the diagnostic criteria and symptomatology of these overlapping diseases, highlighting the complexity involved in accurately differentiating and diagnosing between PANS and PANDAS according to the type and presence of neuropsychiatric signs and their temporal connection with *Streptococcus* infections.

Most multi-disciplinary clinics that are focused on treatment for PANS/PANDAS utilize a three-pronged treatment strategy that exactly parallels the approach used in ARF/SC [38]: (1) Clear identification of infections (sinusitis and GABHS infections including throat, perianal, vaginal, skin, etc.) in patients and close-contacts/household members [39]; (2) employment of anti-inflammatories (like NSAIDS or steroid bursts) [40,41,42] or other immunomodulation and [43]; and (3) introduce a standard of care for psychiatric symptoms including cognitive behavioral therapy [44] and psychotropics starting with low doses and slow and careful up-titration [45].

The evidence supports that the pathogenesis of PANS/PANDAS is immune-mediated and likely targets the basal ganglia. Further efforts to characterize the immunological mechanisms are needed (especially in patients with persistent symptoms) to improve treatment strategies. In this narrative review, we aimed to describe the immunological features observed in PANS/PANDAS patients to date. 

## 2. Methods

In April and May 2024, for this narrative paper, a wide literature search was carried out by three of the authors of the present review to disclose relevant studies on various databases (PubMed, Scopus and WOS). The papers were selected including the following keywords: PANS, PANDAS, neuroinflammation, immune-mediated inflammatory diseases and pediatrics. No filter on the publication year was used. The selected studies were rigorously evaluated and reviewed by all authors to recognize works that potentially encounter the main aim of this narrative review.

Key criteria for the inclusion were *(i)* studies in English, *(ii)* original articles that dealt with the differential diagnosis of PANS and PANDAS and *(iii)* original studies analyzing the descriptions of PANS and PANDAS. Editorials, case reports, and letters were excluded from this narrative review. The papers that met the inclusion and exclusion criteria were further examined, and pertinent data were obtained and evaluated for each study. Any divergences between the authors of the present work were resolved by a consensus approach.

## 3. PANS/PANDAS: Immunological Mechanisms

### 3.1. Th17 Lymphocytes and Cytokine Profiles Including IL-17

In recent years, much attention has been given to the possible role of inflammatory and immunologic alterations in the pathophysiology of OCD and related conditions, such as PANDAS and TS. Dysfunctions of the basal ganglia are common findings throughout this spectrum of neuropsychiatric syndromes. In some of these, such as SC, the autoimmune etiology is well demonstrated [32,33,34,35], while in others such as PANS/PANDAS it is strongly suspected [29,30].

Peripherally circulating and intrathecally produced autoantibodies that bind to synaptic and other neuronal proteins appear to play a role in a spectrum of autoimmune central nervous system (CNS) disorders [46]. There are several proposed mechanisms by which pathogenetic autoantibodies enter the CNS. Recently, the possible role of a specific subtype of lymphocytes, T helper 17 and relative cytokines, in BBB disruption has been investigated as they allow for the entry of cross-reactive antistreptococcal antibodies into the basal ganglia through a process of molecular mimicry. Th17 cells represent a population of lymphocytes involved in the immune response to extracellular infectious agents. Due to their cytokine profile and ability to recruit other immune cell types, they are highly pro-inflammatory and are involved in the induction of many autoimmune disorders [47,48]. Pathogenetic mechanisms include the direct and indirect effects of IL-17 on brain cells [49]. IL-17 is the main Th 17 cytokine.

Circulating IL-17 provokes BBB disruption by altering tight junctions (TJs) and cell-adhesion molecule expression on endothelial cells [50]. In the normal BBB, mononuclear cells penetrate the BBB by way of diapedesis through the cytoplasm of the endothelial cells, without TJ disruption. During inflammatory processes, cytokines may open the TJ between endothelial cells and mononuclear cells may pass by a paracellular process too [51,52].

Disruption in the BBB allows for the entry of peripheral immune cells, including Th17 cells, monocytes, and neutrophils, into the CNS. Moreover, IL-17 prevents brain endothelial cells from producing NO (eNOS), which may lead to endothelial dysfunction and lower cerebral blood flow (CBF) [49]. In the CNS, IL-17 has direct effects on neurons and oligodendrocytes [53]. In vitro studies have revealed that IL-17 blocks the differentiation and survival of oligodendrocyte lineage cells [54]. In a different study, IL-17 induced a reduction in oligodendrocytes via tumor necrosis factor (TNF-α) activity which inhibited their progenitor cell differentiation. Additionally, IL-17 signaling in microglia and astrocytes produces further inflammatory cytokines which promote neutrophil chemotaxis and accumulation. Moreover, this amplifies the production of reactive oxygen species (ROS) by nicotinamide adenine dinucleotide phosphate (NADPH) oxidase and xanthine oxidase, as indicated in vivo and in vitro studies in human brain-derived endothelial cells [55,56]. NADPH oxidase (NOX) is a multisubunit enzyme complex, that plays a key role in several biological functions.

At the cellular level, NOX2 has been reported to be expressed in neurons and astrocytes and is heavily expressed in microglia, where it is involved in inflammatory responses [57]. A recent study suggests that increased oxidative stress, particularly a NOX2 over-activation, may favor the onset and the persistence of the disease in PANDAS patients, since soluble NOX2-dp (sNOX-2-dp), iso-PGF2α and lipopolysaccharide (LPS) were elevated in 30 patients relative to controls. Moreover, the study points to a potential role of gut-derived LPS in eliciting systemic NOX2 levels in children affected by PANDAS, suggesting a potential role for the gut microbiota as a source of oxidative stress in this population [58].

It is, therefore, believed that the Th17 response plays a crucial role in those pathological conditions characterized by a state of neuroinflammation as shown by studies on autoimmune encephalitis (AE) [54] and multiple sclerosis (MS) [59], in which CSF IL-17A levels are increased and correlated with BBB injury [60].

Recent studies have attempted to investigate the role of Th17 lymphocytes in the pathogenesis of PANS/PANDAS. Dileepan developed a mouse model of post-GABHS basal ganglia encephalitis through intranasal infections with live streptococcus. In this model, after repeated intranasal infections, Streptococcus-specific Th17 cells proliferated in the nasal lymphoid tissue and migrated into the brain (via the olfactory nerve path) causing BBB disruption and microglial activation [27]. The repeated intranasal infections expand Th17 cells and shift their cytokine profile to IL-17A+IFN-γ+ [61,62], which are the main cytokines involved in BBB disruption in vitro and in vivo through the generation of ROS in endothelial cells [55,56] known to be found in the CNS in both human MS and rodent models of MS [63]. Murine nasal-associated lymphoid tissue (NALT) is functionally equivalent to human palatine tonsils; thus, the authors further identified streptococcus-specific Th17 cells in human tonsils [61]. Furthermore, streptococcus-specific T-cells were found in the mouse brains 56 days after infection, a latency that coincides with the occurrence of neurological symptoms of chorea, which typically emerge several weeks or months after acute infection. Platt later confirmed the critical role of Th17 lymphocytes in a mouse model of post infectious AE, triggered by multiple infections with GABHS. Furthermore, mice that lack Th17 lymphocytes experience a decrease in BBB leakage, microglial activation, antibody infiltration into the CNS and a partial recovery of olfactory functions [64].

The immunopathogenesis involved in the TH17 GABHS PANDAS mouse model suggests a localized inflammatory process between the nasopharyngeal pathway to the brain. Human data that support this model include a recent study showing a trend toward higher IL-17 levels in the blood of patients with PANDAS compared to controls [65]. Despite the lack of statistical significance in this blood study, a consistent body of evidence suggests that Th17 and IL-17A play a role in the development of many CNS diseases [49]. In patients with GABHS-associated PANS/PANDAS, a lack of elevated IL-17 in the blood does not discount the possibility of IL-17/TH17 cells in the brain, as in other CNS autoimmune conditions [59]. 

However, it should be noted that, despite the evidence currently available in the literature, the association between Th17 cells, IL-17 and PANS/PANDAS remains controversial. To definitively ascertain this connection, further studies are necessary, particularly those involving experimental protocols that embrace a larger cohort of recruited individuals. These investigations should aim to provide more conclusive and comprehensive findings to either refute or support the suggested immunological relationships underlying PANS/PANDAS.

### 3.2. OCD and Cognitive Impairments: Possible Role of Monocytes and Cytokines Other than IL17 in the Neuroinflammatory Circuitry

Currently, the immuno-inflammatory hypothesis of OCD is one of the most interesting and promising research areas. Increased levels of circulating pro-inflammatory monocytes were described in children with OCD suggesting a potential role of monocytes in pediatric-onset OCD anxiety and depression following prolonged stress exposure [66,67]. Additionally, patients with OCD (compared with controls), have elevated IL-1β, IL-6 and TNF-α l. Interestingly the neuropsychological assessment “Trail Making Test A and B” (TMT) which measures attention, mental flexibility and visual and motor speed was positively correlated with IL-6 and TNF-α [68] and the performance on these tests was worse in patients vs. controls. However, other studies reported lower IL-6 and TNF- α levels in OCD patients compared with healthy controls [69]. The state of the OCD condition (new-onset, recovering, chronic-static) likely plays a role in differential findings of biological studies.

Published data on inflammatory markers in PANS patients are scarce. Singer did not find significant differences between patients (mixed-stage) and controls in terms of serum levels of IL-4, IL-10, IL-12 and TNF-α. Furthermore, no correlation was identified between clinical exacerbations of a limited set of autoimmune markers during flare and recovered phases [70]. A more recent study showed higher levels of IgM, TNF-α and IL1-β in a cohort of patients with persistent PANS compared to a non-persistent group [71]. None of the other measured cytokines, IL-6, IL-8 and IL-10, were elevated in this study [71]. Interestingly, none of the patients in this cohort had elevated IL-1-β and/or TNF-α levels at baseline [11]. In line with these results, other studies in the literature demonstrated higher levels of TNF-α during psychiatric exacerbations. Leckman analyzed the serum concentrations of nine cytokines in 11 PANDAS patients (a subgroup of 46 patients presenting with OCD + tics) during 2 years by analyzing serum specimens every 4 months and during exacerbations. In this study, IL-12 and TNF-α concentrations at baseline were elevated in patients compared with control subjects and both were increased during flares compared to recovered state [72]. 

In another study by Parker-Athill, a panel of nine cytokines was assessed in twenty-one children with a chronic tic disorder, including five PANDAS patients. The authors, similarly, reported that 77% of the patients expressed significantly higher levels of serum TNF-α during tic exacerbations compared to improved states [73]. However, in this study, the authors measured TNF- α in children with PANDAS/ADHD/OCD by combining the data together and there is no info on the single groups.

Immunological findings in tic disorders may also inform PANS/PANDAS research given the overlapping symptoms and circuitry. A recent study investigated cytokine levels “mild tics” and “above moderate tics” based on Yale Global Tic Severity Scale (YGTSS) scores for comparison. In the mild tic group, IL-12 p70 negatively correlated with motor tic scores. Granulocyte-macrophage colony-stimulating factor, IL-4, IL-8 and TNF-α were positively correlated to phonic tic scores. IL-12 p40 and TNF-α were positively correlated to total tic scores. Interestingly only patients with mild symptoms exhibited significant correlations. This study also revealed that patients with mild tic symptoms showed lower IL-1β levels than those of healthy controls [74].

### 3.3. Intestinal Inflammation and Immune Response: Possible Implication of Gut Brain Axis in PANDAS/PANS

Several studies in animals and humans have suggested that alterations to the gut microbiota are associated with neuro-inflammation and could have possible implications for neuropsychiatric disorders [75,76,77]. A recent study showed the presence of an altered microbiota in PANDAS/PANS patients (pre-antibiotic treatment) compared to controls, suggesting that GABHS could alter the gut microbiota through the selection of bacterial strains associated with intestinal inflammation and activation of the immune response [78]. According to the gut–brain axis model, an altered bacterial community in the gut may influence behavior, as observed in PANDAS/PANS patients. Additionally, since patients with ARF/SC, PANDAS and post-GABHS PANS often receive antibiotics, alteration of the intestinal flora by antibiotic cannot be dismissed as playing a role in delayed emergences of behavioral symptoms or persistence of symptoms [79].

### 3.4. Arthritis, Autoimmune Disease and Systemic Inflammatory Signs in Youth with PANS

Recent studies suggest that patients with PANS/PANDAS develop arthritis (enthesitis-related arthritis, psoriatic arthritis and spondyloarthritis) and other autoimmune diseases (thyroiditis, celiac disease, etc.) [10,80]. IL-17 has been demonstrated to play a role in the types of arthritis that commonly follow the onset of PANS with a high frequency of immune activation signs (low C4, periungual redness and swelling, prominent onychodermal bands, etc.) at psychiatric illness presentation, suggesting nonspecific autoimmunity and small vessel vascular involvement. The findings of these systemic signs suggest that PANS may be a brain response to a global inflammatory process, rather than an isolated psychiatric disorder [10,80].

Joint capsule thickening noted on joint ultrasound was a common finding among patients with PANS. Joint ultrasonography was routinely ordered in this study to improve the sensitivity and specificity of detecting arthritis given that joint symptoms are often overshadowed by severe psychiatric symptoms and sensory dysregulation in the PANS population. The capsular thickening in the joints may also be a response to a more global inflammatory process [10,80].

### 3.5. Hypogammaglobulinemia in a Subset of PANS/PANDAS Patients

Several studies have investigated the possible role of immunoglobulin deficiency as a factor in recurrent GABHS infections. A study of youth with OCD (including nine PANDAS patients), showed reduced IgA plasma levels (median 115 mg/100 mL) compared to non-PANDAS cases (*n* = 10) and healthy controls (*n* = 12) [81]. The authors speculated that mucosal immunity may be compromised in these patients and that IgA could modify GABHS-driven immune responses. However, no patients in the population analyzed had a diagnosis of absolute IgA deficiency [81]. Another cohort of twenty-seven PANS patients was followed for a median of 3.3 years and elven out of the twenty-seven patients showed an IgG sub-class deficiency, while only one patient had low total IgG and six patients had low IgA levels [71]. Another study of twenty-six patients (relapsing-remitting course of PANDAS) indicated no difference in immunoglobulin levels, IgG subclasses and lymphocyte subpopulation when compared to another vulnerable population (youth with recurrent pharyngitis) [65]. Only one patient had an isolated IgA deficiency and one patient had a lower IgM value for age reference [65].

### 3.6. Autoantibodies in PANS/PANDAS

A spectrum of autoantibodies has been reported in PANS/PANDAS [6]. The most rigorous study into the autoantibodies in PANS/PANDAS which have been shown to have functional consequences involving the basal ganglia is led by Dr. Pittenger at Yale University utilizing samples from five different cohorts to date. His studies, which examined sera from patients with PANS/PANDAS, revealed higher levels of autoantibody binding to cholinergic interneurons (CINs) compared to the controls [29,30]. This binding declines in parallel with symptom improvement after treatment (pre- vs. post-intravenous immune globulin treatment) [82]. Furthermore, the binding of autoantibodies to CINs reduced CIN functional activity, thus disrupting the role of these CINs on the cortico-striatal circuitry. Although CINs express D2 dopamine receptors, it is not yet known whether the autoantibodies that bind CINs do so by targeting dopamine receptors. When these same CINs are experimentally depleted in mice, the striatum becomes hyperactive and the mice develop repetitive behaviors like the symptoms of PANDAS/PANS [83,84].

Molecular mimicry involving antibodies directed against the dominant streptococcal group A carbohydrate epitope (N-acetyl-beta D-glucosamine) which cross-react with neural cells has been proposed by a number of investigators. Pavone et al. detected anti-basal ganglia antibodies in 64% of 22 PANDAS patients compared to 9% of 22 control individuals with an uncomplicated active GABHS infection [85]. Anti-neuronal autoantibodies in PANS/PANDAS patients have been described towards tubulin, lysoganglioside GM1 and dopamine receptors (DR) [31,32,33,34,35,36]. Five groups of human dopamine receptors divided into two classes are described: D1-like (which includes D1R and D5R) and D2-like (which includes D2R, D3R and D4R). These receptors are mainly located in the striatum, the substantia nigra and the hippocampus. Antibodies like anti-D1R/D2R internalize receptors causing dopamine dysregulation. Increased activity of calcium/calmodulin-dependent protein kinase II (CaMKII) also causes a change in dopamine release [86]. The presence of autoantibodies in PANS/PANDAS serum and cerebrospinal fluid (in patients vs. controls) supports the neuroinflammatory hypothesis of this disease.

Chain et al. reported high levels of anti-neuronal autoantibodies and high activity of CaMKII in serum and cerebrospinal fluids of 32 PANDAS patients during the acute symptomatic phase. It is important to notice that some healthy controls also had high antibody levels, but no one had a significant increase in CaMKII activity; therefore, this protein could play a specific role in the neuroinflammation process. Interestingly, during follow up a lower autoantibody titer was detected in patients with clinical improvement [31].

The Cunningham Panel is a laboratory test used in PANDAS/PANS patients which includes antibodies direct against D1R, D2R, Lysoganglioside-GM1 and Tubulin. It also analyses the activity of CaMKII. A retrospective study highlighted the improvement of neuropsychiatric symptoms in 58 PANDAS/PANS patients with a lower autoantibody and CaMKII activation after treatment suggesting that the Cunningham Panel might be a useful tool for follow-up of PANDAS patients [87] but this panel needs further validation. The clinical use of the Cunningham Panel in diagnosing PANDAS/PANS is not supported in the Bejerot et al. study [88], thus more research is needed to determine the diagnostic utility of the Cunningham panel [89].

### 3.7. Immunomodulatory Treatment Trials Lacking in All Pediatric Neuroinflammatory and Many Pediatric Rheumatological Diseases

A recent systematic review of treatments for PANDAS and PANS has shown that rigorously conducted research (i.e., clinical trials) is scarce [82], as is the case for autoimmune encephalitis and many pediatric rheumatological conditions, since clinical trials are very expensive and require significant government and drug company investment which is not yet available to the PANS/PANDAS and AE fields of research. Further research is needed in which promising treatment strategies for PANDAS and other variants of OCD with proposed autoimmune etiology will be investigated.

In consideration of the immunological mechanisms underlying the pathogenesis of PANDAS/PANS, the use of NSAIDs has been proposed to improve psychiatric symptoms [43]. Two observational studies (retrospective assessment of the duration of flares) on PANS patients following treatment with or without NSAID and with or without corticosteroid bursts indicated that these anti-inflammatory treatments may shorten the duration of PANS flare ups akin to ARF/SC [41]. Clinical trials testing NSAIDS and corticosteroids in ARF, SC, PANS/PANDAS have not been conducted to date, yet these treatments remain first-line treatments for these disorders.

Another ex adiuvantibus line of reasoning that supports the autoimmune-inflammatory etiopathogenesis could emerge from the reduction in IgG binding to CINs and improvement in symptoms (in a subset of patients) after infusions of immunoglobulins in PANS/PANDAS patients [29]. Since intravenous immunoglobulin (IVIG) therapy is costly and has conflicting trial results [15,90], it is not universally accessible even though a subset of patients may respond robustly. 

### 3.8. OCD in Neuroinflammatory Disorders

As mentioned previously, there are other diseases characterized by neuroinflammatory injury that have overlapping symptoms with PANS/PANDAS. SC is a late infective complication of GABHS, mediated by an injury to the basal ganglia, but the onset tends to be more sub-acute and chorea is more prominent than the subtle choreiform movements and basal ganglia soft signs seen in PANS/PANDAS [25]. In addition to involuntary movements, patients with SC often have OCD (starting before, during, or after chorea) and prominent behavior changes [6,91]. The pathogenesis is due to a molecular mimicry process that induces autoantibody formation against basal ganglia–thalamocortical circuits, so PANDAS and SC may share an underlying pathogenesis [32]. In SC patients, the inflammatory brain injury can be visible on an MRI with abnormal T1 and T2 signals in the basal ganglia [92] and swelling in the basal ganglia in the acute stage [21] which is like PANS/PANDAS [17,18,19,20].

Epidemiological studies suggest a link between GABHS infection and OCD and TS [93,94], though it is unclear if these studies included patients who met PANDAS/PANS criteria. Regardless, immune dysregulation has been implicated in TS (elevated pro-inflammatory cytokines: IL-17-A, IL-6, IL-12, TNF-α and activation of T&B cells) [95]. Inflammation is also thought to play a role in garden-variety OCD and other anxiety disorders [96]. Lastly, patients diagnosed with “idiopathic movement disorders” were found to have anti-basal ganglia antibodies [97]. 

Elevated levels of CAMKII activity were found in patients with autism spectrum disorder and the presence of anti-tubulin and anti-D2R antibodies (which are antibodies found in SC and PANDAS) were associated with clinical response after IVIG treatment. These studies further suggest that neuropsychiatric disorders manifesting with OCD may have overlapping immune mechanisms [98].

## 4. Conclusions

Recent investigations into PANS and PANDAS reveal diverse strands of evidence implicating post-infectious inflammation, autoimmunity and altered basal ganglia circuitry in their pathogenesis. Numerous studies underscore the differences in immune parameters in patients with these disorders and healthy controls. Significant and enduring immunological alterations post-infection, including elevated autoantibodies and inflammatory responses (pro-inflammatory cytokine profile), and the development of arthritis and other autoimmune conditions have been reported in PANS/PANDAS patients.

However, there are often challenges in differentiating populations of patients with OCD, eating restriction, TS and SC from PANS/PANDAS, especially when the timeline/history is not clear or if neurological symptoms do not squarely fall into one category. However, differentiating these disorders (which are certainly on a spectrum rather than being distinct) may eventually become moot once there are clear markers that guide treatment decisions based on biology rather than downstream symptoms.

However, it should be noted that the concept of PANS/PANDAS is still highly controversial within the biomedical community, mainly because of the unpredictability of the diagnostic criteria, the overlap of the symptoms with other neuropsychiatric diseases and the incomplete knowledge of the fundamental pathophysiological mechanisms [99,100,101]. Critics debate that the existing indications are insufficiently robust, with many works relying on limited sample sizes and missing rigorous controls [102,103]. Nevertheless, the mounting body of immunological and clinical evidence suggests a plausible link between immune dysregulation and neuropsychiatric symptoms, requiring further analyses. From a biomedical perspective, it is critical to approach this pediatric condition with an open mind. More exhaustive, multicenter investigations to corroborate innovative insights into neuroimmune connections are mandatory to improve patient outcomes.

There is a clear need for research to identify reliable clinical biomarkers of active inflammation in these disorders vs. resolved inflammation in the setting of neurological injury (which would guide rehabilitation as opposed to anti-inflammatories). Biomarkers are also required to improve the selection of patients for rigorous clinical trials. Future research should focus on etiological factors (including genetic, infectious, and immunologic) to further elucidate the biological basis of PANDAS and PANS and to refine interventions and strategies for the prevention of relapses.
